# Stress Induced Transcription Factors Transactivate the Herpes Simplex Virus 1 Infected Cell Protein 27 (ICP27) Transcriptional Enhancer

**DOI:** 10.3390/v13112296

**Published:** 2021-11-17

**Authors:** Jeffery B. Ostler, Clinton Jones

**Affiliations:** Department of Veterinary Pathobiology, College of Veterinary Medicine, Oklahoma State University, Stillwater, OK 74078, USA; jostler@okstate.edu

**Keywords:** herpes simplex virus 1, infected cell protein 27 (ICP27) promoter/enhancer, glucocorticoid receptor (GR), Krüppel-like factor 15 (KLF15), cooperative transactivation

## Abstract

Following acute infection, herpes simplex virus 1 (HSV-1) establishes lifelong latency in neurons, including sensory neurons within trigeminal ganglia. During latency, lytic cycle viral gene expression is silenced. However, stressful stimuli can trigger reactivation from latency. The viral tegument protein, VP-16, transactivates all immediate early (IE) promoters during productive infection. Conversely, cellular factors are expected to trigger viral gene expression during early stages of reactivation from latency and in non-neuronal cells that do not support high levels of productive infection. The glucocorticoid receptor (GR), synthetic corticosteroid dexamethasone, and certain stress-induced transcription factors cooperatively transactivate infected cell protein 0 (ICP0) and ICP4 promoters. Since ICP27 protein expression is required for productive infection, we hypothesized that the ICP27 promoter is transactivated by stress-induced transcription factors. New studies have demonstrated that ICP27 enhancer sequences were transactivated by GR and Krüppel-like factor 15 (KLF15). Mutation of a consensus Sp1 binding site within ICP27 enhancer sequences impaired transactivation by GR and KLF15. Chromatin immunoprecipitation studies have demonstrated that GR and KLF15 occupy ICP27 promoter sequences during productive infection. Cells transfected with an ICP27 enhancer fragment revealed the GR and KLF15 occupancy of ICP27 enhancer sequences required the intact Sp1 binding site. Notably, GR and KLF15 form a feed-forward transcription loop in response to stress, suggesting these cellular factors promote viral replication following stressful stimuli.

## 1. Introduction

Herpes simplex virus 1 infection continues to be one of the most prevalent viral infections of the eye [1] Infections can lead to serious recurrent eye disease, including herpetic stromal keratitis (HSK). Corneal scarring and neovascularization are hallmarks of HSK, and chronic HSK can culminate in blindness. Most HSK cases are due to reactivation from latency [2]. However, oral acyclovir treatment only reduces recurrent HSK approximately 50% of the time [3]. Identifying cellular and viral factors that trigger reactivation from latency may serve as valuable targets designed to reduce the incidence of reactivation from latency.

Sensory neurons in the trigeminal ganglia (TG) and other neurons are sites for life-long latent infections following infection of oral, ocular, or nasal cavities [4]. In contrast to productive infection, abundant viral gene expression is not readily detected during latency. The only viral gene abundantly expressed in latently infected neurons is the locus encoding the latency-associated transcript (LAT) [4,5,6,7]. LAT expression plays an important role in reactivation from latency in small animal models of latency [8,9,10,11]. HSV-1 reactivation from latency is essential for virus transmission and recurrent disease. Notably, stressful stimuli correlate with an increased incidence of reactivation from latency in humans [12,13,14,15,16]. Corticosteroids bind to the cytosolic glucocorticoid receptor (GR) and mineralocorticoid receptor (MR) [17,18]. The MR or GR corticosteroid complex is then translocated to the nucleus, where it specifically binds glucocorticoid response elements (GREs) and activates transcription. Notably, de novo protein synthesis is not required for GR or MR activation.

Dexamethasone (DEX) inducible cellular factors in TG neurons were previously identified during early stages of bovine herpesvirus 1 (BoHV-1) reactivation from latency [19] and following explant of mouse TG [20]. A subset of these cellular genes consists of four members of the Krüppel-like factor (KLF) transcription family, for example, KLF4, KLF6, KLF15, and PZLF (promyelocytic leukemia zinc finger). KLF family members belong to the Sp1 super-family of transcription factors [21,22]. Due to the complex nature of glucocorticoid signaling pathways, dissecting the role of these transcription factors through in vitro tissue culture systems has provided important insights into GR and KLF-mediated activation of herpesvirus promoters. The Sp1 transcription factor binds and transactivates HSV-1 immediate early (IE) promoters [23]. KLF family members also can bind certain GC-rich consensus Sp1 binding sites (GGGCGG), suggesting specific KLF family members transactivate HSV-1 IE promoters [21,22]. Previous studies demonstrated GR and KLF15 cooperatively transactivate the HSV-1 ICP0 promoter [24]. Furthermore, GR and KLF4 or KLF15 cooperatively transactivate the HSV-1 ICP4 promoter [25]. GR and KLF15 synergistically activate gene expression by a positive feed-forward transcription loop [26,27,28]. With respect to this positive feed-forward transcription loop, GR stimulates KLF15 expression, leading to GR and KLF15 protein–protein interactions, which culminate in the synergistic activation of certain genes. Since viral genes necessary for productive infection are not abundantly expressed in latently infected sensory neurons, it is reasonable to predict that GR and certain stress-induced KLF family members stimulate key viral promoters during the early stages of reactivation from latency. We further predict stressful stimuli facilitate viral gene expression and productive infection in certain non-neuronal cells. This prediction is supported by the finding that HSV-1 replication is stimulated by DEX in mouse neuroblastoma cells (Neuro-2A) [24].

The multifunctional HSV-1 IE protein ICP27 is required for productive infection [29] because it interacts with numerous host proteins or RNAs and regulates gene expression (viral and cellular), cell cycle, immune responses, and stress responses (reviewed in [30]). Similar proteins are also encoded by additional α-herpesvirinae subfamily members, such as the varicella zoster virus IE4, [31], bovine herpesvirus 1 bICP27 [32], and equine herpesvirus 1 UL3 [33]. Furthermore, the beta-herpesvirus human cytomegalovirus UL69 [34] and gamma-herpesvirus Epstein–Barr virus EB2/SM [35] encode ICP27-like proteins. The most well studied functions of the ICP27 family of proteins are the inhibitions of host gene expression, while promoting virus gene expression. Widespread disruption of transcription termination is characteristic of HSV-1 infection [36], and ICP27 inhibits 3′ end processing of host RNA, including polyadenylation and intron splicing, which are critical steps in RNA post-transcriptional regulation [37,38,39]. ICP27 binds the tegument protein virus host shutoff (VHS), an endoribonuclease that degrades host mRNAs but does not target viral RNAs made during infection. VHS activity is reduced in ICP27-deficient viruses [40,41]. Interestingly, ICP27 binds the characteristically GC-rich HSV-1 RNAs not degraded by VHS [42]. In addition, bICP27 interferes with the activity of beta-interferon 1 and 3 promoters [43]. In contrast, ICP27 promotes virus gene expression, shuttling the generally intron-less virus mRNAs to the cytoplasm [44,45,46,47].

The objectives of this study were to test whether stress induced transcription factors and GR transactivate HSV-1 ICP27 promoter-enhancer sequences, identify sequences necessary for transactivation, and test whether these cellular transcription factors occupy ICP27 promoter-enhancer sequences. The rationale for examining the ICP27 promoter is the multi-functional ICP27 protein is essential for productive infection [29,30].

## 2. Materials and Methods

### 2.1. Cell Cultures

Mouse neuroblastoma cells (Neuro-2A, ATCC CCL-131) and monkey kidney epithelial cells (Vero, ATCC, CCL-81) were grown in MEM with 2 mM L-glutamine, 100 mg/mL Streptomycin, 10 U/mL penicillin, and 10% fetal bovine serum (FBS), as previously described [24,25]. Activated charcoal selectively removes lipophilic molecules, including corticosteroids that drive cellular stress responses, without affecting salts, glucose, or amino acids. This allows for improved control over glucocorticoid receptor activation with specific concentrations of dexamethasone. Neuro-2A cells were also grown in MEM containing 2% or 0.5% charcoal-stripped FBS and, where indicated, 20 µM retinoic acid (Sigma; St Louis, MO, USA; R2625). Serum starvation and retinoic acid both readily induce neurite formation of Neuro-2A cells in culture [48], which is defined as differentiation. To achieve optimal neurite formation, approximately 4 × 10^5^ Neuro-2A cells were plated in a 60 mm dish.

As a comparison to reduced FBS, Neuro-2A cells were cultured in Neurobasal™ medium (Gibco; Birmingham, MI, USA; 21103049) containing B-27 plus supplement (Gibco, Birmingham, MI, USA; A3582801), a specialized medium for neuronal cell culture, following manufacturer’s guidelines.

### 2.2. Virus Infection

The McKrae strain of HSV-1 was obtained as a kind gift from Steve Wechsler and cultured as previously described [49]. For infection studies, Neuro-2A and Vero cells were washed with phosphate buffered saline (PBS), and the virus was added to the cells at an MOI of 1. Cells were incubated for one hour with periodic shaking, washed again with PBS, and further cultured in media containing 2% charcoal stripped FBS. Water soluble DEX (Sigma; St Louis, MO, USA; D2915; 10 μM final concentration) was added where noted. At indicated time points, cells were formaldehyde crosslinked and harvested for ChIP, described below.

### 2.3. Primers

**Figure 1:**
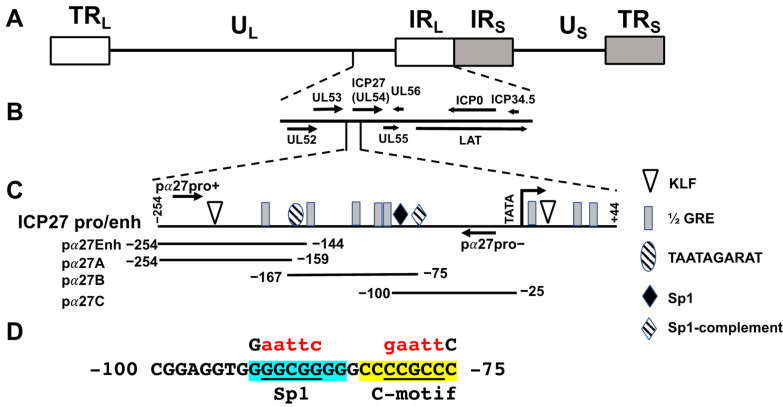
Location of ICP27 gene and promoter within the unique long region of the HSV-1 genome. Panel (**A**): Schematic of HSV-1 genome. Unique long (U_L_) and unique short (U_S_) segments are flanked by the long repeats (R_L_: white rectangles) and short repeats (R_S_: gray rectangles). Repeats are denoted as terminal (T) or internal (I). Panel (**B**): Location of ICP27 (UL54) gene in U_L_ is shown, along with flanking genes in U_L_ and R_L_. Panel (**C**): Schematic of ICP27 promoter, with potential transcription factor binding sites relative to the starting site of transcription (arrow): ½ GREs (grey rectangle), potential KLF binding sites (white triangle), Sp1 binding site (black diamond), and TAATAGARAT motif (striped oval). Four enhancer fragments (pα27Enh, −254 to −144; pα27A, −254 to −159; pα27B, −167 to −75; pα27C, −100 to −25) used in this study are shown. These fragments were cloned upstream of pGL4.24[luc2/minP] firefly luciferase reporter plasmid (Promega; Madison, WI, USA). Positions of pα27 pro+ and − PCR primers are indicated. These primers yield a 191 bp PCR product. Panel (**D**): Shared nucleotides of pα27B and pα27C fragments (−100 to −75). Sp1 binding site (blue highlighted bases) and C-rich motif containing Sp1 complementary site (yellow highlighted bases) are indicated. Mutated bases are in red and underlined in wt sequences.

**Figure 2:**
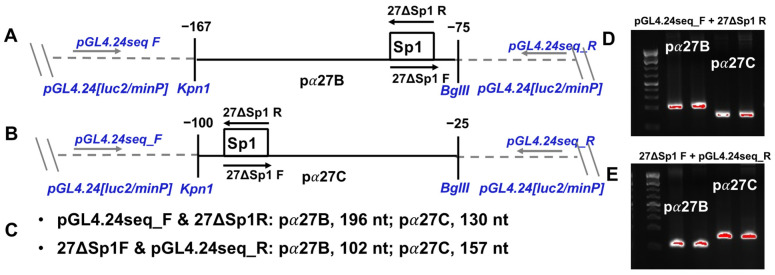
Schematic of ICP27 enhancer fragments in luciferase reporter plasmid and position of PCR primers used for ChIP. The pα27B (Panel (**A**)) and pα27C (Panel (**B**)) enhancer fragments cloned into pGL4.24[luc2/minP] upstream of the minimal promoter. Enhancer fragments, denoted by black lines, are shown with nucleotide positions relative to the transcriptional initiation site. Sp1 binding site (white box) is shown in the approximate location of each enhancer fragment, along with the primer pair used for mutagenesis, 27ΔSp1-F and -R. Luciferase reporter plasmid pGL4.24[luc2/minP] (grey dashed line) is shown with *Kpn1* and *BglII* restriction sites used to insert enhancer fragments. Approximate positions of pGL4.24 seq-F and -R primers indicated. Panel (**C**): Primer pairs used to PCR amplify ChIP DNA and size of PCR products generated from each primer pair and enhancer fragment. Panels (**D**,**E**): PCR product from the indicated primer pair on each enhancer fragment, visualized on a 2% agarose gel stained with ethidium bromide.

Primers were used for amplification of viral DNA, ICP27 enhancer fragments, and generating Sp1-mutant enhancer fragments. Three primer sets were used in these studies, with name, sequence, and location of target DNA listed below. The ICP27 forward (pα27 pro+) and reverse (pα27 pro-) primers were used to amplify virus genomic DNA from infected cells, generating a 190 nt product (Figure 1C). The ICP27ΔSp1 forward (27ΔSp1_F) and reverse (27ΔSp1_R) were used to create Sp1 binding site mutants by PCR-directed mutagenesis and to amplify pα27B and pα27C enhancer fragments, in combination with the pGL4.24 seq primers (Figure 2A–C). Nucleotides in bold are different from template sequences, creating the Sp1 binding site mutant fragments. pGL4.24[luc2/minP] forward (pGL4.24 seq_F) and reverse (pGL4.24 seq_R) primers were used to sequence Sp1 binding site mutants. These were also used to amplify pα27B and pα27C enhancer fragments in combination with the ICP27ΔSp1 primers: pGL4.24 seq_F with 27ΔSp1_R and 27ΔSp1_F with pGL4.24 seq_R. Both primer pairs amplified wild-type and mutant pα27 B and pα27C enhancer fragments, with distinctive sized PCR products (Figure 2D,E). 

The pα27 pro+ primer is 5′-CCGTCCCGTTACCAAGAC-3′ (nucleotides −237 to −220 of the ICP27 promoter). The -pα27 pro-primer is 5′-TCTCCCAACCCCTCCTC-3′ (nucleotides −64 to −47 of the ICP27 promoter).

The 27ΔSp1_F primer is 5′-CGGAGGTGGAATTCCGGCCCC-3′ (nucleotides −101 to −80 of the ICP27 promoter, mutant Sp1 binding site underlined). The 27ΔSp1_R primer is 5′-CGGGGCCGGAATTCCACCTCC-3′ (nucleotides −79 to −99 of the ICP27 promoter, mutant Sp1 binding site underlined).

The pGL4.24 seq_F primer is 5′-ACATACGCTCTCCATCAAAAC-3′ in the pause site, 5′ of multiple cloning site, and 99 nt 5′ of Kpn1 insertion site (Figure 2A,B), spanning nt 4320 to nt 4341 of pGL4.24[luc2 P/minP]. The pGL4.24 seq_R primer is: 5′-ACAGTACCGGATTGCCAA-3′ between the minP and luciferase coding sequence, 81 nt 3′ of BglII insertion site (Figure 4A,B), spanning nt 109 to nt 127 of pGL4.24[luc2 P/minP].

### 2.4. Plasmids

A pGL3-Promoter plasmid containing the HSV-1 strain F ICP27 enhancer element, pα27Enh, spanning nucleotides −254 to −114, cloned between MluI and XhoI restriction sites, was generously provided by Thomas Kristie (NIH; Bethesda, MD, USA). Nucleotide positions are relative to the transcription initiation site. The enhancer element was released from pGL3 and cloned into pGL4.24[luc2P/minP] (Promega; Madison, WI, USA) at unique KpnI and BglII sites upstream of the minimal promoter driving expression of a firefly luciferase gene. pGL4.24[luc2/minP] was chosen as the firefly luciferase reporter plasmid because numerous cryptic transcription factor binding sites were removed. Three additional enhancer elements were synthesized by GenScript and inserted into the pGL4.24[luc2P/minP] plasmid at the same site: pα27A (nucleotides −254 to −159), pα27B (nucleotides −167 to −75), and pα27C (nucleotides −100 to −25, Figure 1C). Sp1 binding site mutants of pα27B and pα27C were generated by PCR-directed mutagenesis: the Sp1 binding site (GGGCGG) in both plasmids was mutated by replacing the core sequence with an EcoRI restriction site (GAATTC), as shown in (Figure 1D). The C-motif and Sp1/C-motif double mutants were synthesized by GenScript in the pGL4.24[luc2P/minP] plasmid (Figure 1D). A mouse GR-α expression plasmid was obtained from John Cidlowski (NIH), and the KLF15 expression plasmid was obtained from Deborah Otteson (University of Houston; Houston, TX, USA).

### 2.5. PCR-Directed Mutagenesis

The Sp1 binding site (GGGCGG) in the pα27B and pα27C enhancer fragments was mutated by the QuikChange^TM^ site directed mutagenesis method (Stratagene; La Jolla, CA, USA) [50,51]. The 27ΔSp1_F and 27ΔSp1_R primers (see primers section) with a three-nucleotide mismatch in the Sp1 binding site (GAATGG) were used to PCR amplify the enhancer-containing plasmids grown in *E. coli* (DH5α, Zymo; Irvine, CA, USA; T3007). A proofreading polymerase, Pfu (M774A, Promega; Madison, WI, USA), was used to reduce second-site mutations. The restriction enzyme Dpn1, which selectively digests methylated DNA, was used to remove template DNA isolated from *E. coli*, leaving only PCR-amplified mutant plasmid. This sample was transformed into *E. coli*, isolated by alkaline lysis, and purified with cesium chloride [52,53]. Resultant pα27BΔSp1 and pα27 CΔSp1 plasmids were sequenced to confirm the Sp1-site mutation and ensure that no secondary site mutations were present. Mutant plasmids were used for dual luciferase and ChIP assays.

### 2.6. Dual Luciferase Assay

Neuro-2A and Vero cells were cultured in 60 mm dishes until 80% + confluency. Two hours before transfection, cells were washed with PBS and antibiotic free media with 2% charcoal stripped FBS added. Neuro-2A cells grown in Neurobasal™ medium were washed with PBS and new media were added before transfection. Neuro-2A cells cultured in reduced levels of stripped FBS (0.5 or 2%) were washed 48 h following initial incubation with stripped FBS, and new, antibiotic free media were added to dishes. Cells were transfected with pGL4.24[luc2/minP] luciferase reporter plasmid containing one of the enhancer fragments upstream of the minimal promoter (0.5 µg DNA, Figure 1C) and with a plasmid expressing Renilla luciferase (0.05 µg DNA), from a minimal TK promoter as a transfection control. Where indicated, GR-α (1 µg DNA) and KLF15 (0.5 µg DNA) expression plasmids were co-transfected with the reporter plasmid. Empty vector plasmid was added as needed to maintain equal quantities of DNA in all transfection reactions. Transfection was carried out using TransIT-X2 (Mirus; Madison, WI, USA; MIR6003) following manufacturer’s instructions. At 24 h following transfection, water soluble DEX (Sigma; St Louis, MO, USA; D2915; 10 μM final concentration) and RU486 (Sigma; St Louis, MO, USA; M8046; 10 μM final concentration) were added to designated samples. At 48 h post transcription, cells were washed, harvested using passive lysis buffer, and stored at −80 °C. Lysates were subjected to a dual luciferase assay, as previously described [24,25]. Firefly and Renilla luciferase activity in each sample were measured using a commercially available kit (Promega; Madison, WI, USA; E1910) and a Glomax 20/20 luminometer (Promega; Madison, WI, USA; E5331). Promoter activity in the pGL4.24[luc2/minP] luciferase reporter plasmid was calculated as a ratio of the reporter firefly luciferase to Renilla luciferase activity, the transfection control. Activity of the empty pGL4.24[luc2/minP] plasmid transfected alone was set as the promoter baseline. Increased promoter activity over this baseline was calculated as fold activation by the enhancer elements and transactivation by GR and KLF15.

### 2.7. Chromatin Immunoprecipitation

Neuro-2A and Vero cells for ChIP were cultured in 100 mm dishes until ~80% confluency. At 2 h prior to transfection or infection, cells were washed with PBS and antibiotic free media with 2% stripped FBS added. Cells were transfected with pGL4.24[luc2P/minP] plasmid (4 µg DNA) containing wild-type (pα27 B, pα27 C) or mutant (pα27 BΔSp1, pα27 CΔSp1) ICP27 enhancer elements, using TransIT-X2 (Mirus; Madison, WI, USA; MIR 6003) according to manufacturer’s instructions. Where indicated, cells were co-transfected with GR-α (3 µg DNA) and KLF15 (3 µg DNA). At 24 h post transfection, designated samples were treated with DEX (10 μM). At 48 h post transfection, cells were formaldehyde crosslinked and harvested for ChIP.

Transfected cells were used for ChIP, as previously described [25,54,55]. Cell lysate was sonicated to shear DNA and cleared with agarose beads and salmon sperm DNA to reduce non-specific binding. Cleared samples were immunoprecipitated using GR-α (5 µg) or KLF15 (5 µg) specific antibodies, with non-specific IgG (5 µg) as an isotype control. Precipitated DNA was amplified by PCR using 27ΔSp1 and pGL4.24 seq primers (Figure 2A–C). Amplified DNA was separated on 2% agarose gel stained with ethidium bromide for visualization. Bands were quantified with ImageLab software (Biorad; Hercules, CA, USA;), and data were presented as a % of the input sample, which represents approximately 13% of cleared cell lysate.

Infected cells were used for ChIP following the same protocol. Precipitated DNA from these samples was PCR amplified using pα27 pro primers (Figure 1C).

## 3. Results

### 3.1. Identification of Enhancer Sequences in the ICP27 Promoter

The HSV-1 ICP27 gene is located in unique long sequences, adjacent to the inverted repeats (Figure 1A,B). The 5′ upstream promoter/enhancer sequences contain several transcription factor binding sites, including TAATGARAT, KLF, Sp1, and ½ GREs (Figure 1C). These ICP27 enhancer sequences are crucial for transactivation by VP-16 and host cell factor 1 (HCF-1), and HCF-1 occupies these sequences during early stages of explant-induced reactivation from latency [56,57,58]. Initial studies revealed that an ICP27 promoter/enhancer construct was modestly transactivated by GR-α (hereafter referred to as GR) and KLF15. Since this construct was cloned in a vector that contains many cryptic transcription factor binding sites, four ICP27 enhancer fragments were prepared and then inserted upstream of the pGL4.24[luc2/minP vector: pα27Enh, pα27A, pα27B, and pα27 C (Figure 1C). This luciferase vector has few binding sites for cryptic transcription factors and encodes a luciferase protein with a short ½ life. Consequently, the effects of stress and stress-induced transcription factors should be readily detected in this reporter construct [20,25]. 

**Figure 3:**
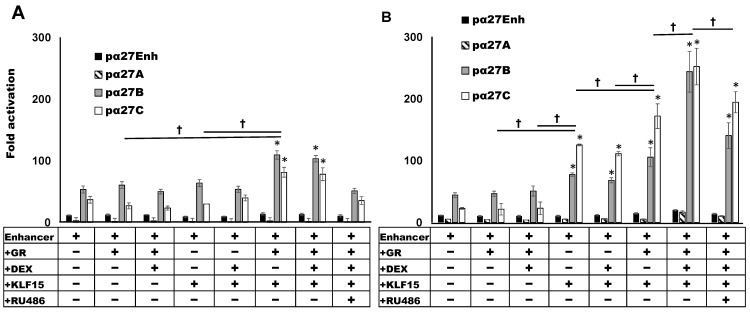
Transactivation of ICP27 enhancer activity by GR and KLF15. Neuro-2A (Panel (**A**)) or Vero (Panel (**B**)) cells were cultured in MEM containing 10% FBS. Two hours prior to transfection, media were replaced with MEM containing 2% charcoal stripped FBS. Cells were transfected with pGL4.24[luc2P/minP] luciferase reporter plasmid containing the designated enhancer fragment (0.5 µg DNA) and a *Renilla* luciferase expression plasmid (0.05 μg) as a transfection control. Where indicated, cells were co-transfected with the GR (1 µg DNA) and/or KLF15 (0.5 µg DNA) expression plasmid. Empty vector was added as needed to maintain equal amounts of DNA in each reaction. Certain cultures were treated with DEX (10 µM) or RU486 (10 µM) 24 h following transfection. At 48 h post transfection, cells were harvested, and luciferase assays performed as described in the materials and methods. Promoter activity was calculated as a ratio of firefly to *Renilla* luciferase activity. Fold activation in each sample is relative to the pGL4.24[luc2P/minP] minimal promoter alone. Results are the mean of three separate experiments, and error bars indicate the standard error. An asterisk (*) denotes statistical significance between the indicated sample and the enhancer alone. A dagger (**†**) indicates significant differences between designated treatment groups for pα27B and pα27C enhancer fragments, as determined by Student’s *t*-test (*p* < 0.05).

To test whether the ICP27 enhancer constructs activate a heterologous promoter and are transactivated by stress-induced transcription factors, Neuro-2A and Vero cells were transfected, and promoter activity measured. Neuro-2A cells were used for these studies because they are neuronal-like cells that can be differentiated into dopaminergic-like neurons [59]. Neuro-2A cells can also be readily transfected, whereas human neuronal cells we use are not readily transfected. Monkey kidney Vero cells are highly permissive to HSV-1 infection and are a good comparison to Neuro-2A cells. In Neuro-2A cells, the pα27B (grey column) and pα27C (white column) constructs stimulated minimal promoter activity by 53- and 38-fold, respectively (Figure 3A). The pα27Enh construct stimulated minimal promoter activity by 10-fold (Figure 3A, black column) in Neuro-2A cells, while the pα27A fragment exhibited low levels of basal enhancer activity (Figure 3A striped column). The ICP27 enhancer constructs had similar effects in transfected Vero cells (Figure 3B).

GR or GR and DEX did not dramatically increase ICP27 enhancer constructs in Neuro-2A or Vero cells. However, KLF15 significantly increased pα27B and pα27C promoter activity in Vero cells, but not Neuro-2A cells. Furthermore, DEX treatment did not significantly increase promoter activity. When pα27B or pα27C were co-transfected with GR and KLF15, promoter activity was significantly increased in Neuro-2A and Vero cells, relative to the basal enhancer activity or when the luciferase construct was transfected with GR or KLF15. Notably, DEX treatment further increased promoter activity in Vero cells but not in Neuro-2A cells. The corticosteroid antagonist RU486 [60,61] significantly reduced the effects of GR and KLF15 on pα27B and pα27C in Neuro-2A cells, despite DEX not significantly increasing promoter activity (Figure 3A). In Vero cells, RU486 reduced pα27B or pα27C promoter activity to levels observed when co-transfected with GR and KLF15 (no DEX treatment). In summary, these studies revealed GR and KLF15 synergistically transactivated the pα27B or pα27C in Neuro-2A and Vero cells. Cell-type differences were observed with respect to the effects of DEX on GR and KLF15-mediated transactivation.

### 3.2. Influence of Neurite Formation in Neuro-2A Cells on ICP27 Enhancer Activity

**Figure 4:**
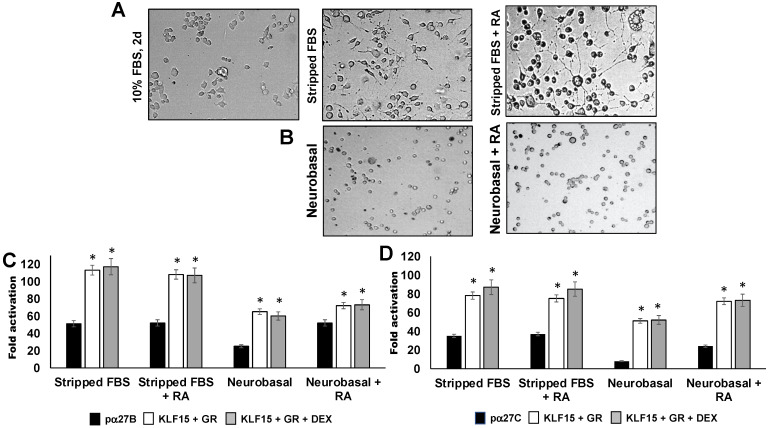
Effect of serum starved neurite formation and Neurobasal™ media on ICP27 enhancer activity. Panel (**A**): Neuro-2A cells were incubated in 10% FBS, 2% stripped FBS, or 2% stripped FBS and RA for 48 h. Neuro-2A cells were seeded at 4 × 10^5^ cells per 60 mm dish to allow for optimal neurite formation. Panel (**B**): Neuro-2A cells were incubated in Neurobasal™ medium with B-27™ plus supplement for 48 h. Live cell images were captured 48 h after cells were plated using an EVOS XL Core imaging system (Life Technologies; Carlsbad, CA, USA; AMEX1200). Representative images are presented (40 x magnification). Panel (**C**): Neuro-2A cells were transfected with pα27B (0.5 µg DNA) alone or with GR (1.0 µg DNA) and KLF15 (0.5 µg DNA) expression plasmids. Panel (**D**): Neuro-2A cells were transfected with pα27C (0.5 µg DNA) alone or with GR (1.0 µg DNA) and KLF15 (0.5 µg DNA) expression plasmids. For C and D, cultures were grown in 2% stripped FBS or in Neurobasal™ medium with B-27™ plus supplement. RA was added to certain cultures as indicated. *Renilla* luciferase expression plasmid was included as a transfection control, and empty vector added as needed to maintain equal amounts of DNA for each transfection. Certain samples were treated with DEX (10 µM) 24 h following transfection. Luciferase assays were performed as described in the materials and methods, and promoter activity calculated. Fold activation in each sample is relative to the pα27B or pα27C transfected alone. Results presented are the means of three separate experiments, with error bars denoting the standard error. An asterisk (*) signifies a statistically significant difference between the indicated sample and enhancer construct alone (black bar), as determined by the Student’s *t*-test (*p* < 0.05).

Additional studies examined ICP27 enhancer activity in Neuro-2A cells after incubating cultures with MEM containing 2% stripped FBS or Neurobasal™ media containing the B-27 plus supplement. The effects of retinoic acid (RA) on these treatments were also examined, because reducing FBS and retinoic acid consistently arrests the cell cycle and establishes a neuronal-like phenotype in Neuro-2A cells, as denoted by neurite formation [59,62]. Consistent with our previous studies [63,64,65,66], incubating Neuro-2A cells in 2% stripped FBS for 48 h stimulated neurite formation (Figure 4A), which was enhanced by RA treatment. Although Neurobasal™ and a B-27 supplement are generally used for primary rodent neurons, its effects were examined in Neuro-2A cells. Incubation of Neuro-2A cells with Neurobasal™ media containing the B-27 growth supplement and RA addition induced cells to round up and growth was arrested. However, neurite formation was not observed (Figure 4B). 

The pα27B construct was strongly transactivated by GR and KLF15 regardless of DEX treatment, after the Neuro-2A cells were incubated with 2% stripped FBS (Figure 4C). Similar results were obtained when Neuro-2A cells were incubated in 0.5% stripped FBS for 2–4 days regardless of RA treatment (data not shown). When Neuro-2A cultures were incubated in Neurobasal™ and B-27 growth supplement, GR + KLF15-mediated transactivation of pα27B was reduced relative to stripped FBS-mediated differentiation. Interestingly, incubation of Neuro-2A cells with Neurobasal™ medium reduced pα27B basal promoter activity, unless RA was added.

GR and KLF15 significantly increased pα27C promoter activity when cultures were incubated with 2% stripped FBS, but DEX did not significantly change promoter activity (Figure 4D). Consistent with pα27B, incubation in Neurobasal™ media supplemented with B-27 growth supplement reduced pα27C promoter activity relative to 2% stripped FBS. In summary, induction of neurite formation in Neuro-2A cells by incubation with stripped FBS and/or RA did not dramatically alter the ability of GR and KLF15 to mediate transactivation of pα27B or pα27C compared to untreated Neuro-2A cells (Figure 3).

### 3.3. Sp1 Binding Site Mediates GR- and KLF15-Dependant Transactivation

Studies in Figure 3 and Figure 4 suggest ICP27 enhancer sequences located between −100 and −75 contain an important cis-acting element for GR and KLF15 cooperative transactivation. Interestingly, there is a consensus Sp1 binding site (GGGCGG) and a C-rich motif that is a reverse complement of the Sp1 binding site (CCCGCC) located between −100 and −75 (Figure 1D). Furthermore, the GC-rich motif with dyad symmetry (denoted by the light blue and gray shaded nucleotides in Figure 1D) appears to be important for transactivation by GR and KLF15. Certain Sp1 binding sites are bound by KLF family members (25, 54) because KLF and Sp1 family members belong to the same super-family of transcription factors (21, 22). The two ½ GREs are only present in pα27B sequences, suggesting they are not essential for GR and KLF15-mediated transactivation. To assess whether the GC-rich motif that contains a consensus Sp1 binding is important for transactivation by GR and KLF15, the wild-type Sp1 site, GGGCGG, in pα27B and pα27C was mutated to GAATCC, which also disrupts the dyad symmetry of this GC-rich motif (Figure 1D). Vero or Neuro-2A cells were transfected with plasmids containing wild-type (wt) enhancer constructs or the Sp1 binding site mutants (pα27BΔSp1 or pα27CΔSp1), and promoter activity was measured. 

**Figure 5:**
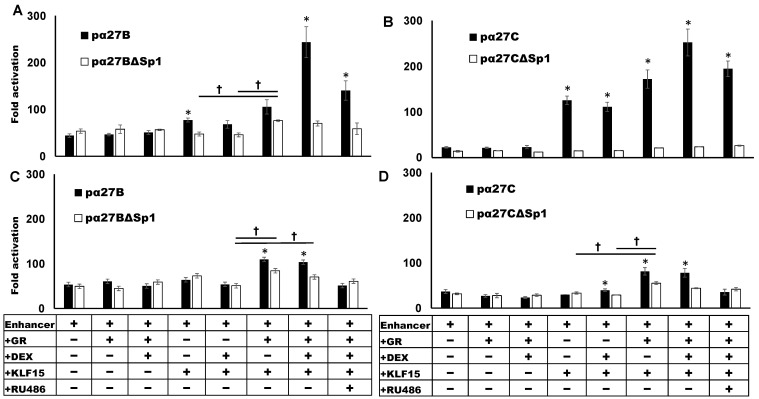
Mutation of Sp1 binding site impairs GR and KLF15-dependent transactivation of ICP27 enhancers. Vero cells (Panels (**A**,**B**)) or Neuro-2A cells (Panels (**C**,**D**)) were cultured in MEM with 2% stripped FBS and transfected with the denoted enhancer fragments (0.5 μg DNA) and a *Renilla* luciferase expression plasmid (0.05 μg DNA) as a transfection control. The reporter plasmid contained either the wt or Sp1-mutant enhancer fragment, pα27B (Panel (**A**)) or pα27C (Panel (**B**)). Where indicated, cells were co-transfected with a GR (1 μg DNA) or KLF15 (0.5 μg DNA) expression plasmid. Empty vector plasmid was added to maintain equal amounts of DNA in each transfection. Cells were treated with DEX (10 μM) or RU486 (10 μM, mifepristone) at 24 h post transfection and harvested at 48 h post transfection. Enhancer activation was measured, and fold activation was calculated. The results represent the mean of three separate experiments, and error bars indicate standard error. An asterisk (*) indicates a significant change in promoter activity between the wild-type and Sp1-null enhancer fragment. A dagger (**†**) denotes a significant difference between indicated samples, as determined by Student’s *t*-test (*p* < 0.05).

Basal enhancer activity levels of pα27BΔSp1 or pα27CΔSp1 were like the respective wt constructs in Vero (Figure 5A,B) and Neuro-2A cells (Figure 5C,D). In the context of pα27B, the Sp1 binding site mutation significantly reduced transactivation by KLF15 alone in Vero cells and cooperative transactivation by GR, DEX, and KLF15. Relative to pα27C, KLF15 and/or GR, regardless of DEX treatment, did not significantly increase pα27CΔSp1 promoter activity in Vero cells. Consistent with studies in Figure 3, DEX treatment increased the effects of transactivation by GR and KLF15 for wt pα27B and pα27C, but not the Sp1 mutants in Vero cells. GR and KLF15-mediated transactivation of pα27BΔSp1 and pα27CΔSp1 in Neuro-2A cells was significantly reduced compared to the respective wt construct. However, the effect was not as dramatic as in Vero cells. Collectively, these studies revealed that a consensus Sp1 binding site was crucial for GR and KLF15-mediated transactivation of the ICP27 enhancer. 

### 3.4. C-Motif Was Not as Important as Sp1 Binding Site for GR and KLF15-Mediated Transactivation

**Figure 6:**
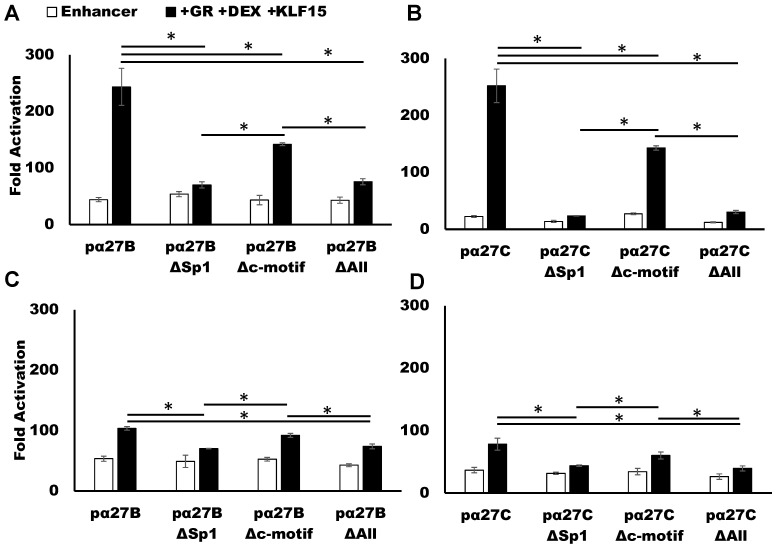
Effects of C-motif mutants on transactivation of ICP27 enhancer. Vero cells (Panels (**A**,**B**)) or Neuro-2A cells (Panels (**C**,**D**)) were cultured in MEM with 2% stripped FBS and transfected with pGL4.24[luc2P/minP] (0.5 μg DNA) and a *Renilla* luciferase expression plasmid (0.05 μg DNA). The reporter plasmid contained wild-type, Sp1 mutant, C-motif mutant, or Sp1- and C-motif mutants, pα27B (Panel (**A**)) or pα27C (Panel (**B**)). Where indicated, cells were co-transfected with a GR (1 μg DNA) and KLF15 (0.5 μg DNA) expression plasmid. Empty vector plasmid was added to maintain equal amounts of DNA in each transfection. Cells were treated with DEX (10 μM) at 24 h post transfection and harvested. Enhancer activation was measured using a dual luciferase assay. Fold activation of each enhancer fragment was calculated. The results represent the mean of three separate experiments, and error bars indicate standard error. An asterisk (*) indicates a significant change in promoter activity between the indicated enhancer fragments with GR, DEX, and KLF15, as determined by Student’s *t*-test (*p* < 0.05).

Additional studies tested whether the C-rich motif adjacent to the Sp1 binding site (Figure 1D) regulated the ability of GR, KLF15, and DEX treatment to cooperatively transactivate ICP27 enhancer sequences in Vero or Neuro-2A cells (Figure 6). pα27B or pα27C reporter constructs containing a wt construct, Sp1 mutant, C-rich motif mutant, or a Sp1- and C-rich mutant were compared to the empty pGL4.24[luc2P/minP] minimal reporter construct. In Vero cells, mutation of the C-motif in pα27B (Figure 6A) and pα27C (Figure 6B) by GR and KLF15 in the presence of DEX was significantly reduced. However, mutating the Sp1 binding site or Sp1 site and C-rich motif significantly reduced promoter activity compared to just mutating the C-rich motif in Vero cells. In contrast to Vero cells, mutating the C-motif did not significantly reduce GR, KLF15, and DEX-mediated transactivation in Neuro-2A cells (Figure 6C,D). As in Vero cells, mutating the C-motif and/or Sp1 binding site significantly reduced GR, KLF15, and DEX-mediated transactivation in Neuro-2A cells. These studies demonstrated that mutating the Sp1 site alone was sufficient to block transactivation. However, the C-rich motif had a modest, but significant, effect on cooperative transactivation in Vero cells. 

### 3.5. ICP27 Enhancer Occupancy by GR and KLF15 Requires an Intact Sp1 Binding Site

**Figure 7:**
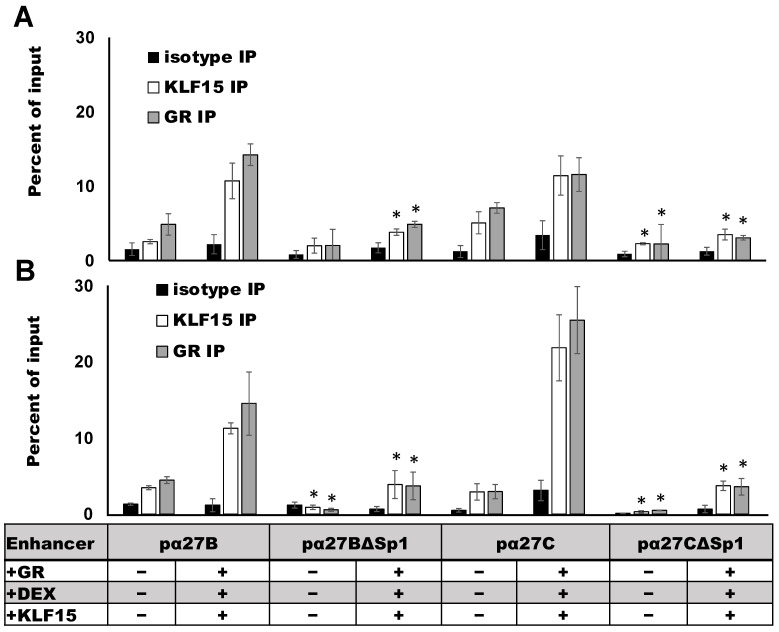
GR and KLF15 occupy ICP27 enhancer sequences. Neuro-2A (Panel (**A**)) and Vero cells (Panel (**B**)) were cultured in MEM containing 2% stripped FBS and transfected with the denoted enhancer constructs (4 μg, Figure 4A). Certain samples were co-transfected with GR (3 μg DNA) and KLF15 (3 μg DNA). Empty vector was added to maintain the same amount of DNA in each transfection. At 24 h post transfection, samples were treated with DEX (10 μM). At 48 h post transfection, ChIP was performed. DNA–protein complexes were immunoprecipitated with GR (5 μg) or KLF15 (5 μg) antibodies. Non-specific isotype IgG was used as a negative control. Immunoprecipitated DNA was purified, amplified by PCR, and run on a 2% agarose gel and then stained with ethidium bromide to visualize the PCR products. Primers for PCR are described in Figure 2C. Relative enrichment of DNA in the antibody samples was measured using image lab software (Biorad; Hercules, CA, USA) and presented as a percentage of input (13.3% of total cell lysate). Data represent the means of 3 separate experiments, and error bars indicate the standard error. Asterisks (*) denote a significant difference in DNA amplification of the Sp1 binding site mutants compared to wt samples, as determined by Student’s *t*-test (*p* < 0.05).

To assess whether GR and KLF15 occupy ICP27 enhancer elements, Neuro-2A and Vero cells were transfected with the wild-type pα27B or pα27C enhancer construct, and chromatin immunoprecipitation (ChIP) was performed using GR- or KLF15-specific antibodies and primers designed to specifically amplify ICP27 enhancer sequences (Figure 2). In Neuro-2A cells, low levels of endogenous GR and KLF15 were associated with pα27 B and pα27C enhancer fragments when transfected with the empty vector (Figure 7A). Overexpression of KLF15 and GR increased the occupancy of both the pα27B and pα27C enhancer sequences. Mutating the Sp1 binding site significantly reduced the occupancy of overexpressed GR and KLF15 for both pα27B and pα27C fragments. When compared to empty vector controls, GR and KLF15 occupancy increased significantly when GR and KLF15 were overexpressed in the presence of DEX in Neuro-2A cells (Figure 7B). Consistent with results in Neuro-2A cells, GR and KLF15 occupancy was significantly reduced by mutating the Sp1 binding site. These studies demonstrated GR and KLF15 occupy the ICP27 promoter. However, mutating the Sp1 binding site significantly reduced GR and KLF15 occupancy. 

### 3.6. ICP27 Promoter/Enhancer Sequences Are Occupied by GR and KLF15 during Productive Infection

**Figure 8:**
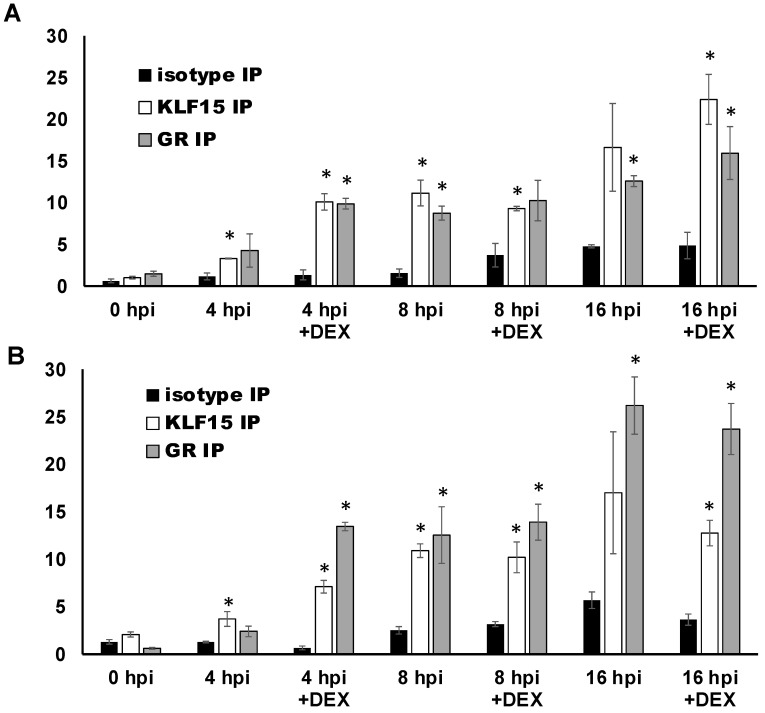
GR and KLF15 occupancy of the ICP27 promoter changes during productive infection. Neuro-2A (Panel (**A**)) or Vero cells (Panel (**B**)) were infected with HSV-1 (McKrae strain) at an MOI of 1 and then cultured in MEM containing 2% stripped FBS. Certain samples were treated with DEX (10 μM). At the indicated times after infection (0, 4, 8, and 16 hpi), ChIP was performed. The 0 hpi timepoint sample was washed with PBS, crosslinked, and harvested following 1 h incubation. ChIP was performed by immunoprecipitation by GR (5 μg) or KLF15 (5 μg) antibodies and non-specific isotype IgG as a negative control. ICP27 promoter DNA was amplified by PCR using pα27 pro +/− primers (Figure 1C), which generates a 191 nt PCR product. PCR products were quantified using Image Lab software. Enrichment of DNA by GR and KLF15 antibodies is presented as a percentage of the input sample (13.3% of total sample). Data shown are the means of 3 independent experiments, and error bars indicate the standard error. Asterisks (*) identify significant differences in DNA amplification between the specific antibody samples and isotype control, as determined by Student’s *t*-test (*p* < 0.05).

To assess whether GR and KLF15 occupy ICP27 promoter sequences during productive infection, Neuro-2A (Figure 8A) and Vero (Figure 8B) cells were infected with HSV-1 and the cells harvested at 0, 4, 8, and 16 h post infection (hpi) for ChIP studies. In Neuro-2A cells at 4 hpi, DEX treatment significantly increased KLF15 and GR occupancy relative to the isotype control (Figure 8A). By 8 hpi, GR and KLF15 occupancy, regardless of DEX treatment, increased to similar levels. GR and KLF15 occupancy of the ICP27 promoter was also readily detected at 16 hpi. However, KLF15 occupancy was only significant relative to isotype in the presence of DEX. In Vero cells, significantly higher levels of GR and KLF15 occupied the ICP27 promoter by 4 hpi when treated with DEX. By 8 hpi, GR and KLF15 occupancy of the ICP27 promoter had increased to the same level as GR with DEX at 4 hpi. However, DEX treatment did not significantly enhance promoter occupancy. GR occupancy increased by 16 hpi, but KLF15 occupancy was unchanged. KLF15 occupancy of the ICP27 promoter at 16 hpi was only significant relative to the isotype control antibody in the presence of DEX. In summary, these studies demonstrated GR and KLF15 occupy ICP27 promoter sequences during productive infection. However, DEX significantly increased promoter occupancy only at 4 hpi. These studies support the prediction that stressful stimuli and KLF15 stimulate viral replication. 

## 4. Discussion

In this report, we demonstrate that ICP27 enhancer sequences were transactivated by GR and KLF15 in Neuro-2A and Vero cells. Although it was clear that GR and KLF15 cooperatively transactivated pα27B and pα27C enhancers, DEX treatment only had a modest effect in Vero cells but essentially no effect in Neuro-2A cells. The GR construct used for these transfection assays expresses the GR-α isoform because it transactivates GRE containing promoters better than the other 7 GR isoforms generated by alternative splicing [67]. Neuro-2A cells express a smaller GR than GR-α, which we suspect is GR-β [54]. GR-β, unlike GR-α, is not a strong trans-activator of promoters following corticosteroid treatment. However, the endogenous GR in Neuro-2A cells clearly binds to the ICP27, ICP0 [24], and ICP4 [25] promoters. Previous studies demonstrated that the immediate early transcription unit 1 (IEtu1) promoter of bovine herpesvirus 1 is transactivated by DEX more efficiently when co-transfected with the GR-α expression construct [54,68]. Two consensus GR response elements (GRE) in the IEtu1 promoter are essential for GR-α and DEX-mediated transactivation. Furthermore, the mouse mammary tumor virus LTR is strongly transactivated by DEX and GR-α expression plasmids in Neuro-2A [65], which is consistent with independent studies [69]. Hence, we believe transactivation of the ICP27 enhancer fragments by GR-α and KLF15 does not require DEX in Neuro-2A cells and only has a modest effect in CV-1 cells. Although this was unexpected, ligand-independent GR activation has been reported [70,71].

Relative to basal enhancer activity, GR or KLF15 alone did not efficiently transactivate the pα27B or pα27C enhancer construct in Neuro-2A cells. However, KLF15 significantly stimulated both enhancer constructs in Vero cells, regardless of DEX treatment. For both cell types examined, GR and KLF15 cooperative transactivation occurred. While these studies suggest cell-type specific factors are important for differences in GR and/or KLF15-mediated transactivation, mutating the Sp1 binding site clearly reduced GR and KLF15 transactivation to basal levels in both cell types. Notably, the Sp1 binding site mutants exhibited little change in basal enhancer activity in both cell lines, suggesting this motif was more important for GR and KLF15-mediated transactivation. Sequences overlapping pα27B and pα27C enhancers (−100 to −75) contain the only Sp1 binding site in the ICP27 enhancer fragment and a C-rich motif with dyad symmetry adjacent to the Sp1 binding site (Figure 1D). While mutating the C-rich motif had a modest effect on GR and KLF15 cooperative transactivation in Vero cells, mutating the Sp1 and C-rich motif had the same effect as mutating the Sp1 binding site. The occupancy of the enhancer fragments by GR and KLF15 was significantly reduced by mutating the Sp1 binding site in both enhancer fragments and in both cell lines, indicating KLF15 interacts with sequences encompassing the intact Sp1 binding site. Thus, the Sp1 consensus binding site and to a lesser extent the C-rich motif are predicted to anchor GR, KLF15, and other transcriptional coactivators to this site. Collectively, these studies provide evidence there is a correlation between KLF15 and GR cooperative transactivation and the ability of these transcription factors to interact with sequences that encompass the Sp1 binding site within ICP27 enhancer sequences. 

Several dissimilar external stimuli coincide with increased reactivation from latency in humans. These stimuli include increased corticosteroid levels, UV light, trauma, and heat stress (fever) [4,5,12,13,16,72,73,74]. Strikingly, certain reactivation stimuli have been reported to activate GR by an unliganded mechanism [75]. For example, ligand independent GR phosphorylation correlates with increased UV exposure [70,76]. Consequently, GR responsive genes have increased mRNA [77]. Secondly, heat stress also activates GR by an unliganded mechanism under certain instances [69]. Cyanoketone, a glucocorticoid synthesis inhibitor, reduces the incidence of heat-stress induced reactivation from latency in a mouse ocular model of HSV-1 infection [14], suggesting that heat stress can activate GR via ligand-dependent or independent mechanisms. Finally, β_2_-adrenergic receptor agonists and, in part, cAMP induce ligand-independent GR activation [78]. The HSV-1 ICP4 enhancer is cooperatively transactivated by GR and KLF4, promyelocytic leukemia zinc finger protein (PLZF), or SLUG [25]. Furthermore, ligand-independent activation of GR and related nuclear hormone receptors also mediates transactivation of the BoHV-1 bICP0 early promoter [52,53,55]. While the mechanism that mediates unliganded activation of steroid receptors is not well understood, published studies have demonstrated that ligand-independent GR phosphorylation modulates certain cellular stress signaling pathways [70].

Stress, in general, increases the frequency of reactivation from latency in humans, as reviewed in [12,13,15,16]. While corticosteroids and GR impair immune responses [17,79], these events are predicted to influence the frequency of reactivation from latency in vivo after stress stimulates viral gene expression and productive infection. This prediction is supported by the finding that HSV-1 explant-induced reactivation from latency [49] and productive infection [24,80] are enhanced by the synthetic corticosteroid DEX. Secondly, GR and stress-induced transcription factors, including KLF15, cooperatively transactivate the ICP0 [24], ICP4 [25], and ICP27 promoters/enhancers. Thirdly, a recent study demonstrated that HSV-1 infection of cultured cells increases KLF15 steady state levels and silencing KLF15 expression significantly reduces viral replication [81]. Since GR and KLF15 form a feed-forward transcriptional circuit to stimulate gene expression [28], we suggest that the GR/KLF15 regulatory axis activates key viral promoters during early stages of reactivation from latency and/or viral spread in non-neuronal cell types following stressful stimuli. The ICP0, ICP4, and ICP27 promoters/enhancers do not contain a consensus “whole” GRE, suggesting that additional HSV-1 promoters are cooperatively transactivated by GR and KLF15 because Sp1 binding sites and other G-rich motifs are present in many viral promoters.

## 5. Conclusions

In conclusion, we believe GR activation, via liganded and un-liganded mechanisms, can increase the frequency of reactivation from latency or viral replication in certain non-neuronal cells. The feed forward transcription loop comprised of GR and KLF15 is also predicted to trigger viral gene expression and replication. Our studies do not rule out the possibility that other reactivation stimuli activate different cellular signaling pathways and transcription factors that trigger viral gene expression during early stages of reactivation. Finally, the ability of GR and stress-induced cellular transcription factors to transactivate key HSV-1 regulatory promoters may activate viral gene expression and productive infection in non-neural cells. Understanding the mechanism by which cellular factors trigger viral gene expression has translation implications. For example, if the essential cellular transcription factors necessary for activating viral gene expression during early stages of reactivation from latency are identified, these cellular transcription factors become potential therapeutic targets.

## Data Availability

Not applicable.
